# High Levels of FGF11 Correlate with Poor Survival in Patients with Human Papillomavirus (HPV)-Positive Oropharyngeal Squamous Cell Carcinoma

**DOI:** 10.3390/cancers15071954

**Published:** 2023-03-24

**Authors:** Caroline Haglund de Flon, Linnea Haeggblom, Stefan Holzhauser, Ourania N. Kostopoulou, Mark Zupancic, Tina Dalianis, Eva Munck-Wikland, Linda Marklund, Anders Näsman

**Affiliations:** 1 Department of Oncology-Pathology, Karolinska Institutet, Bioclinicum J6:20, Karolinska University Hospital, 171 64 Stockholm, Sweden; 2 Department of Clinical Pathology, CCK R8:02, Karolinska University Hospital, 171 64 Stockholm, Sweden; 3 Medical Unit Head Neck Lung and Skin Cancer, Department of Head and Neck Surgery, Karolinska University Hospital, 171 64 Stockholm, Sweden; 4 Department of Clinical Science, Intervention and Technology—CLINTEC Division of Ear, Nose and Throat Diseases, Karolinska Institutet, University Hospital, 171 64 Stockholm, Sweden; 5 Department of Surgical Sciences, Section of Otolaryngology and Head and Neck Surgery, Uppsala University, 751 05 Uppsala, Sweden

**Keywords:** FGF11, oropharyngeal cancer, tonsillar cancer, survival, prognosis

## Abstract

**Simple Summary:**

To better identify patients with human papillomavirus (HPV)-positive oropharyngeal cancer (OPSCC) and a poor prognosis after treatment, we compared the gene expression in tumours from patients with a poor or a favourable prognosis in a case-control setting. The results were thereafter validated in two separate cohorts on the RNA and protein levels. High RNA or protein expression of FGF11 was correlated with a poor patient survival in all three cohorts. Taken together, the data imply that FGF11 may play a major role in the prognosis of patients and that FGF11 could serve as a prognostic marker in HPV-positive oropharyngeal cancer.

**Abstract:**

Human papillomavirus (HPV)-positive oropharyngeal squamous cell carcinoma (OPSCC) is associated with a favourable prognosis. It has therefore been suggested that treatment should be individualized and separated by HPV status. However, additional prognostic markers are still needed before treatment can be individualized for this patient group. For this purpose, all patients diagnosed with HPV and p16-positive OPSCC in Stockholm 2000–2009, identified as having a partial/nonresponse to treatment and having viable tumour cells in their neck specimen with material available were categorized as cases. These were matched to controls (complete responders), and the differences in the gene expression were analysed. Two separate verification cohorts were identified including patients with HPV- and p16-positive OPSCC, and the data from the case-control study were verified by qPCR and immunohistochemistry (IHC) in the respective cohorts. A separation of gene expression in correlation with survival was observed in the case-control study, and FGF11 expression was identified as significantly differently expressed between the two groups. The prognostic role of FGF11 was validated in the two cohorts on the RNA and protein levels, respectively. Taken together, our findings suggest that FGF11 may indicate a poor prognosis in HPV-positive OPSCC and may serve as a prognostic biomarker.

## 1. Introduction

Oropharyngeal squamous cell carcinoma (OPSCC) comprises squamous cell carcinomas of the tonsil (TSCC), the base of tongue (BOTSCC), and of the oropharyngeal walls and soft palate (otherOPSCC) [1]. The global incidence of OPSCC amounts to around 100,000 cases annually, and around 30% of these cases are human papillomavirus-related (HPV+) [2]. Notably, there has been an increase in HPV+ OPSCC in many Western countries, with a continuous increase for the last 50 years without evidence of a slowdown [3,4,5,6,7]. The epidemic rise in OPSCC was first observed in Sweden, with a current incidence of almost 400 new cases per year [4,8,9]. Meanwhile, HPV-independent (HPV−) OPSCC has decreased in many countries, attributed to a decrease in traditional risk factors, e.g., smoking and alcohol consumption [2,4,10].

HPV+ OPSCC has a better prognosis than its HPV− counterpart, with an 80% rather than a 25–50% 5-year disease-specific survival [4,11]. In addition, the HPV+ tumours more frequently present with lymph node metastasis at diagnosis as compared to their HPV− counterparts [12]. Early-stage tumours commonly are treated with surgery or radiotherapy alone; however, treatment of advanced tumours commonly entails neoadjuvant or adjuvant chemoradiotherapy as well as lymph node dissection of the neck if lymph node metastases remain after oncological treatment. These treatments and procedures are often associated with troublesome side effects, and there is a general assumption that some of these patients receive unnecessarily intensive treatment. On the other hand, although patients with HPV+ OPSCC in general have a better survival, prognostic markers are still needed to identify the rare patient population with HPV+ OPSCC and poor clinical outcome, before treatment can possibly be tapered [2,4,11]. 

The aim of this study was therefore to identify biomarkers that could predict the response to treatment and thereby patient survival. For this purpose, patients with HPV+ TSCC classified as non- or partial responders (NR and PR, respectively) to oncological treatment were matched to patients with a complete response (CR), with the aim to find new biomarkers in an explorative microarray analysis setting and to further validate the data in external cohorts of OPSCC patients.

## 2. Materials and Methods

### 2.1. Patients and Tumour Material

All patients diagnosed with HPV-DNA-positive (HPV+) tonsillar and base of tongue squamous cell carcinoma (TSCC and BOTSCC, respectively) in the County of Stockholm, Sweden between 2000 and 2009 were identified from previous studies (*n* = 311) [9,13,14,15]. These patients had all been previously tested for the presence of HPV DNA by PCR and p16^INK4a^ expression by immunohistochemistry (IHC). 

From the 311 patients, 12 cases were selected for the initial test cohort (the microarray expression test cohort, Table 1) based on the following selection criteria: patients diagnosed with a p16^INK4a^ (p16)-overexpressing and HPV-type-16-DNA-positive TSCC, determined as partial or non-responders to treatment with a subsequent neck dissection containing “viable tumour cells” and with available tumour material from their primary pretreatment TSCC biopsy. Matching patient controls diagnosed with TSCC and complete response (CR) and with available pretreatment tumour material were selected and matched for age, stage, WHO performance status (as a proxy of full dose treatment), and treatment (Table 1). 

The response to treatment was obtained from the medical records and was defined by the surgeon three months after the completed treatment, which in all cases consisted of full dose radiotherapy (RT) (conventional or hyperfractionated RT, total dose 68 Gy) and in one case and one control case of concomitant cisplatin treatment. 

To validate the data obtained from the test cohort, two validation cohorts were used. Both consisted of patients diagnosed with OPSCC in 2000–2009, one with available primary tumour material usable for RNA extraction (*n* = 47, Table 2) and the other with available paraffin embedded tissue slides for validation of the data on the protein level (*n* = 44, Table 2).

### 2.2. RNA Extraction

All primary tumour tissue blocks were reviewed by a second pathologist to verify the diagnosis and to select the representative tumour material. RNA was extracted from 60 μm paraffin-embedded primary tumour specimens with the Qiagen RNeasy FFPE kit, following the manufacturer’s instruction. Haematoxylin–eosin-stained slides were collected before and after sectioning of the tissues used for RNA extraction, in order to verify the presence of tumour in the analysed material. 

### 2.3. Affymetrix Microarray Expression Analysis

In total, 50 ng of RNA from each FFPE sample was used to generate amplified and biotinylated sense-strand cDNA from the entire expressed genome according to the GeneChip^®^ WT Pico Reagent Kit User Manual (P/N 703262 Rev 1 Affymetrix Inc., Santa Clara, CA, USA). GeneChip ST Arrays^®^ (GeneChip Equine Gene 1.0 ST Array) were hybridized for 16 h in a 45 °C incubator, while rotating at 60 rpm. According to the GeneChip^®^ Expression Wash, Stain and Scan Manual (PN 702731 Rev 3, Affymetrix Inc., Santa Clara, CA, USA), the arrays were then washed and stained using the Fluidics Station 450 and finally scanned using the GeneChip Scanner 3000 7G. 

### 2.4. Microarray Data Analysis

The raw data were normalized in Expression Console, provided by Affymetrix (http://www.affymetrix.com, accessed on 23 February 2016), using the robust multiarray average (RMA) method that was first suggested by Li and Wong [16,17]. Subsequent analysis of the differences in the gene expression was carried out in the Transcriptome Analysis Console (TAC) Software 4.0 (Affymetrix Inc., Santa Clara, CA, USA). To increase the percentage of variance and to better visualize the patterns of the original dataset, a 3D Principal Component Analysis (PCA)-plot was used to visualize the microarray data.

### 2.5. qPCR

cDNA synthesis was performed utilizing the SuperScript VILO cDNA Synthesis Kit (Invitrogen, Waltham, MA, USA) by following the manufacturer’s instructions. The mRNA expression levels of FGF11 and the housekeeping gene GAPDH were quantified using the TaqMan technology on an ABI PRISM 7500 sequence detection system (PE Applied Biosystems, Waltham, MA, USA). The sequence-specific primers and probes were FGF11 (Hs00182803_m1) and GAPDH (Hs03929097_g1) (Applied biosystems). The delta–delta CT method was used to calculate the relative gene expression.

### 2.6. Immunohistochemistry

Immunohistochemistry (IHC) was performed by manual processing using a standard avidin–streptavidin method as previously described [18]. In short, FFPE sections were deparaffinized and rehydrated followed by antigen retrieval (citrate buffer pH 6.0), and the endogenous peroxidase was blocked. The slides were thereafter incubated in horse serum followed by incubation with the primary monoclonal mouse anti-FGF11 antibody at 4 °C overnight (diluted 1:100, clone MM0282-6J20; Abcam, Cambridge, UK). The sections were then incubated with a biotinylated secondary anti-mouse IgG followed by incubation with an avidin–streptavidin complex (VECTASTAIN Elite ABC-kit, Vector Laboratories, Burlingame, CA, USA) for detection and DAB for visualization. The slides were counterstained with haematoxylin.

### 2.7. Staining Evaluation

The tumour sections and external positive and negative controls (normal tonsillar tissue) were examined through light microscopy by two researchers/surgical pathologists blinded for clinical outcome. In case of disagreement, unanimity was reached through compromise. The percentage of positive tumour cells staining the cytoplasmic compartment was scored and rounded off to the nearest 5%. Cytoplasmic staining intensity of the tumour cells was evaluated using a three-tier scale of weak, intermediate, and strong. The tumour cells were also evaluated for cytoplasmic staining intensity compared to that of the benign adjacent epithelium using a three-tier scale of weaker, equivalent, and stronger. 

### 2.8. Statistics

The chi-square test was used for categorical data and the Student’s *t*-test to compare mean values. A Wilcoxon signed rank test was used to assess the differences in tumour staining compared to the normal adjacent staining.

Survival was measured in years from the date of diagnosis to a defined event or to five years after diagnosis, when patients were censored. An event was defined as death due to any cause (overall survival, OS), death with OPSCC present (disease-specific survival, DSS), or recurrence in OPSCC (disease-free survival, DFS). Differences in survival were tested using the log-rank test. The Kaplan–Meier estimator was used to estimate the DFS, DSS, and OS. 

All calculations were performed using the IBM SPSS Statistics for Mac (IBM Corp. Released 2021. IBM SPSS Statistics for Macintosh, Version 28.0. Armonk, NY: IBM Corp.).

## 3. Results

### 3.1. Patients and Their Characteristics

The patients included in the test cohort and their characteristics are depicted in Table 1. In short, no differences between the patients’ clinical characteristics were observed, except for the response to treatment (CR vs. PR/NR). The patients included in the validation cohorts and their clinical characteristics are presented in Table 2.

### 3.2. Gene-Expression Analysis

The initial explorative gene-expression analysis was performed on the test cohort described in Table 1. In total, 2222 transcript clusters were significantly (*p* = 0.05) differentially expressed (1002 upregulated and 1220 downregulated) between primary tumours from patients with CR (controls) and patients with PR/NR (cases). However, no transcript clusters were found to be significantly differentially expressed (FDR *p*-value 0.05) after adjusting for multiple testing. 

**Figure 1:**
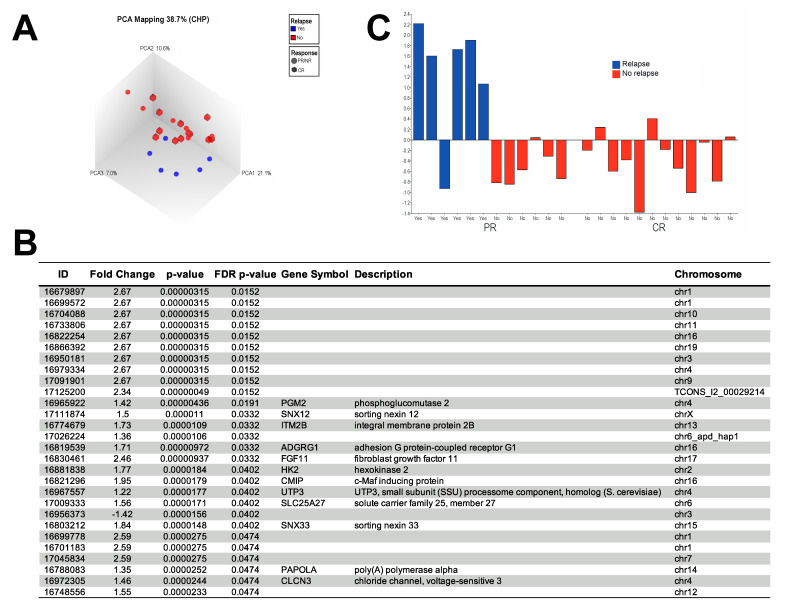
(**A**) Principal component analysis (PCA) plot representing the differentially expressed genes in patients with favourable versus poor survival and complete response versus partial/non-response to treatment. Here, poor survival was defined as a locoregional recurrence within 5 years, and favourable survival was defined as alive and disease free >5-years after diagnosis. Notably, a tendency to separation was observed between patients with (blue) and without (red) relapse in disease. No separation was observed between patients defined as non-/partial responders to treatment (circles) and complete responders (boxes). (**B**) The list of differentially expressed genes (FDR < 0.05) in patients with favourable versus poor survival. (**C**) Bar chart showing the relative tumour FGF11 gene expression as determined by microarray analysis in all 24 patients. Patient bars are arranged according to treatment response and colour coded according to relapse status.

After reviewing the Principal Component Analysis (PCA) plot of all tumours from patients with PR/NR and the matched tumours from patients with CR, no separation could be observed between these groups. However, when reanalysing the patient data, six patients with PR/NR had a locoregional recurrence and died of disease within five years after the primary diagnosis (defined here as poor diagnosis). In contrast, the other six patients with PR/NR stayed disease-free and were all alive more than five years after diagnosis (defined here as favourable prognosis), similar to all patients diagnosed with CR. Nevertheless, now, a tendency to separation in the PCA plot was noticed if poor prognosis was considered instead (Figure 1A), which may suggest that tumours from patients with poor prognosis were more similar than tumours from patients with PR/NR. Therefore, in the further analyses, we focused on differences in the gene expression between tumours from patients with favourable prognosis compared to those with poor survival. In that analysis, 4988 transcript clusters were significantly (*p* = 0.05) differentially expressed (2725 upregulated and 2263 downregulated, Table S1), and after adjusting for multiple testing (FDR *p*-value 0.05), 28 transcript clusters (27 upregulated and 1 downregulated) were found to be differentially expressed (Figure 1B).

When applying a filtering value of *p* = 0.01 and a fold change of at least +/−2, 79 transcript clusters were identified as differentially expressed in patients with favourable versus poor survival (Supplementary Table S1). However, only 14 transcripts were significantly expressed (FDR *p*-value = 0.05) after adjusting for multiple testing. Here, *FGF11* was identified as the only transcript cluster encoding gene with a public gene ID (Figure 1B,C). Therefore, the subsequent analyses focused on *FGF11* expression only and its correlation with prognosis. 

### 3.3. Validation of the FGF11 RNA Levels as a Prognostic Marker by qPCR

**Figure 2:**
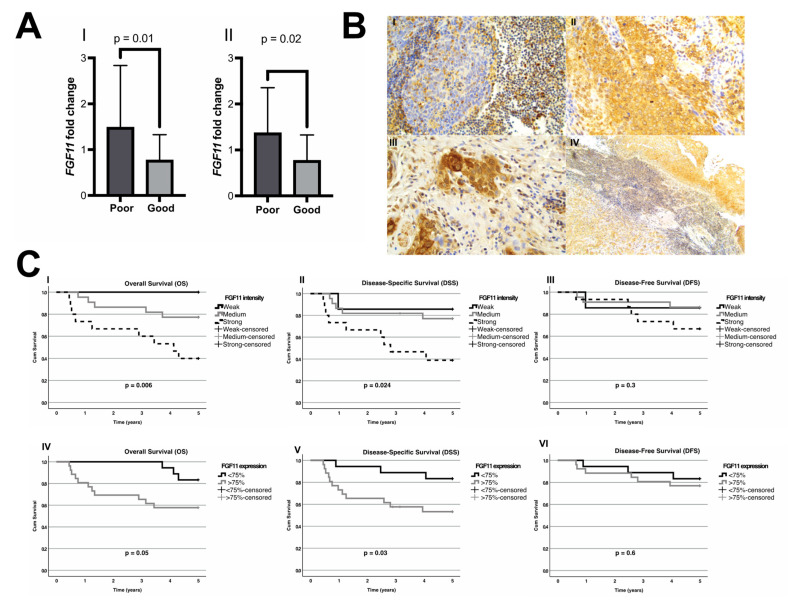
(**A**) Bar graphs (including standard deviation, SD) showing a significant (*p* = 0.01 and *p* = 0.02, respectively) difference in the relative *FGF11* gene expression between good and poor survival as determined by qPCR. Plot I: poor survival defined as a locoregional recurrence and/or dead with disease. Plot II: poor survival defined as a locoregional recurrence. (**B**) Microphotographs of examples of FGF11-immunohistochemical staining and their interpretation. I, top left: absence of FGF11 staining in tumour cells with surrounding positive lymphoepithelial tissue and tumour infiltrating lymphocytes. II, top right: moderate cytoplasmic immunoreactivity in tumour cells. III, bottom left: strong cytoplasmic immunoreactivity in tumour cells. IV, bottom right: weak cytoplasmic immunoreactivity in tumour cells, also weaker than in the adjacent normal epithelium. (**C**) Survival curves depicting overall survival (OS), disease-specific survival (DSS), and disease-free survival (DFS) with regard to FGF11 tumour staining intensity (plots I–III) and the proportion of tumour cells expressing FGF11 (plots IV–VI). Patients with a strong FGF11 intensity had a significantly poorer OS and DSS (*p* = 0.006 and *p* = 0.024, respectively). Similarly, patients with a stronger FGF11 intensity tended to have a worse DFS, compared to patients with weak intensity; however, these differences were not statistically significant (*p* = 0.3). Moreover, patients with a higher fraction of FGF11 positive cells (>75%) had a significantly worse OS and DSS as compared to patients with a lower fraction of positive tumour cells (*p* = 0.05 and *p* = 0.03, respectively). However, no differences were observed in the DFS between these two patient groups (*p* = 0.6).

In order to validate the prognostic value of *FGF11* RNA expression in OPSCC, a separate cohort of 47 patients diagnosed with HPV-positive TSCC and BOTSCC with available RNA was identified and selected. The patients’ characteristics are depicted in Table 2. In this cohort, a high expression of *FGF11* was significantly associated with a worse clinical outcome. In more detail, patients with HPV+ OPSCC with a locoregional recurrence and/or dead with disease within 5 years after diagnosis had significantly higher *FGF11* levels (*p* = 0.01, Figure 2A). Likewise, if only locoregional recurrence was considered as an event in this cohort, patients with a locoregional recurrence had significantly higher FGF11 levels, as compared to all other patients (*p* = 0.02, Figure 2A).

### 3.4. Validation of FGF11 as a Prognostic Protein Marker by Immunohistochemistry

To further validate the prognostic role of FGF11 expression on the protein level, FFPE slides were obtained from 44 patients diagnosed with HPV-positive OPSCC and stained for FGF11 by immunohistochemistry. The immunoreactivity for FGF11 in the TSCC (*n* = 9) and BOTSCC (*n* = 35), as well as the adjacent normal epithelium, was evaluated by two surgical pathologists. FGF11 was predominantly detected in the cytoplasm of the neoplastic and the normal cells, but the FGF11 expression level, as measured by staining intensity, was significantly different compared to that in the adjacent normal epithelium (*weaker n* = 4, *equal n* = 10, and *stronger n* = 30; *p* < 0.0001, Figure 2B).

To investigate the prognostic role of the FGF11 expression, the expression and intensity of the immunoreactivity to FGF11 was compared to the patients’ overall, disease-specific, and disease-free survival (OS, DSS, and DFS respectively). Notably, in general, the protein expression data confirmed the RNA expression data, in which a stronger tumour FGF11 immunoreactivity was associated with a worse survival. More specifically, patients with a strong intensity of cytoplasmic FGF11 immunoreactivity in their tumours had a significantly worse OS (44%) as compared to those with a weak tumour reactivity (100%) (log rank, *p* = 0.006, Figure 2C). The corresponding figures for the DSS and DFS were 39% vs. 86% (log rank, *p* = 0.024) and 67% vs. 86% (log rank, *p* = 0.3), respectively (Figure 2C). In addition, patients whose tumours had a weaker FGF11 immunoreactivity than the adjacent normal epithelium were all alive at followup (*n* = 4). However, the difference in survival was not significant when comparing patients with weaker tumour to normal epithelium FGF11 expression to those patients with a stronger tumour to normal epithelium FGF11 expression. 

Likewise, when comparing the fraction (percentage) of FGF11 positive tumour cells, patients with a higher fraction of FGF11 positive cells (>75%) had a significantly worse OS, compared to those with fewer positive tumour cells (58% vs. 83%, log rank, *p* = 0.047). The corresponding figures for the DSS and DFS were 53% vs. 83% (log rank, *p* = 0.03) and 77% vs. 83% (log rank, *p* = 0.6), respectively (Figure 2C).

## 4. Discussion

In this study, we identified FGF11 as a prognostic marker in OPSCC by first using an explorative gene-expression setup, followed by a verification of its prognostic role on both the RNA and protein level in two separate cohorts of OPSCC patients. Taken together, the data imply an important role of FGF11 for the prognosis of patients with HPV+ OPSCC. 

The initial “case-control” setup included patients with PR/NR with viable tumour cells in their neck specimen defined as “cases” that were matched with regard to age, stage, and treatment to patients with CR to treatment defined as controls. Due to the overall good response to treatment in patients with HPV+ OPSCC, the cases were rare in the total cohort, and a 10-year period was needed to collect a sufficient number of cases. However, during this time period, treatment schemes for this cohort changed (from conventional RT only to more hyperfractionated RT with/without concomitant chemotherapy). Therefore, the WHO performance status data were also obtained from medical charts and used as a “proxy” for full dose treatment. Nevertheless, no significant differences between the patients with NR/PR and CR were observed; therefore, we argue that the two different study groups were similar with regard to all the studied parameters, with the exception of response to treatment (CR, PR/NR). 

Notably, no separation in RNA expression was observed in the PCA plot between tumours from patients with CR and NR/PR, which may suggest that there is a biological heterogenous process behind the diagnosis of response to treatment. Another partial explanation to why no separation was observed may be that the micromorphological assessment of viable tumour cells is difficult for the pathologist and has a low sensitivity and specificity. However, in the subgroup analysis a tendency of separation was observed in the PCA plot, which may suggest a more biological homogenous process. 

Few transcripts were identified as significantly differently expressed between the survival groups in the subgroup analysis, and *FGF11* RNA expression was identified as the only prognostic transcript encoding a public gene ID. Furthermore, its prognostic role in HPV-related OPSCC was further verified by two different methods in two separate cohorts. Initially, we identified *FGF11* expression as significantly differentially expressed between patients that had a locoregional relapse and died with their disease within five years, and this finding was further validated in a separate cohort by qPCR. However, the protein levels of FGF11 were not correlated with the DFS in the second validation cohort, which we assume may partially be explained by the limited number of patients and the few events in that cohort. 

Fibroblast growth factors (FGFs) comprise a large family of cell signalling proteins involved in cell proliferation, survival, migration, invasion, and angiogenesis. They have been shown to have an oncogenic role in many cancer types; however, FGF signalling may also have tumour suppressing capacities [19]. The large family of FGF conserved signalling proteins are therefore notably divided into three groups of proteins: (i) the intracellular FGFs, (ii) the hormone-like (endocrine) FGSs, and (iii) the canonical (paracrine) FGFs [20]. 

FGF11 belongs to the intracellular group and is therefore not secreted and is located within the cytoplasm. Intracellular FGFs are suggested to function as intracellular proteins independent of fibroblast growth factor receptors (FGFRs); irrespectively, previous studies have shown the importance of FGF11 for tumour growth, invasion, and metastatic potential [20,21,22]. A recent study evaluated the role of FGF11 in non-small cell lung cancer (NSCLC), and the authors suggested that FGF11 functions as an oncogene in NSCLC tumour progression [23]. Another study showed that the expression levels of FGF11 increased by hypoxia, and it has also been reported to decrease the degradation of HIF-1α [24]. Notably, increased levels of HIF-1α have been associated with a worse prognosis in many types of cancer, including OPSCC [25]. It is possible that the negative impact of FGF11 levels on the prognosis observed here is mediated by variations in the levels of HIF-1α, but further studies would be needed before such a conclusion could be drawn. 

We acknowledge some limitations of this study. The survival and clinical data were collected retrospectively from patient records. However, of note, all data were collected prospectively in the patient records during their regular follow ups. In addition, the number of patients in all cohorts were limited, and the number of events was not compatible with performing a multivariable analysis. Consequently, the data presented above should be considered as only exploratory, and in order to clearly determine the prognostic impact of FGF11, its correlation with survival should also be investigated in a randomized prospective setting. 

## 5. Conclusions

In summary, this study shows that overexpression of FGF11 could indicate poor survival in patients with HPV-positive OPSCC. Furthermore, the data imply that FGF11 may play a major role in the prognosis of patients with these tumours. We therefore hypothesize that FGF11 could potentially be of clinical use as a prognostic biomarker and could potentially be of use for better tailoring patient treatment. However, prior to this, further studies are needed to disclose the molecular role of FGF11 in OPSCC.

## Figures and Tables

**Table 1:** Patients included in the first test set and their tumour characteristics.

		Patients with HPV+ TSCC and PR/NR ^1^	Patients with HPV+ TSCC and CR ^1^	*p*-Value	Total
**Sex**	Male	9 (75%)	8 (67%)	1.0 ^2^	17 (71%)
	Female	3 (25%)	4 (33%)		7 (29%)
**Age**	Mean	57.8 years	57.4 years	0.9 ^3^	57.6 years
	Min	42 years	43 years		42 years
	Max	74 years	72 years		74 years
**Tumour size TNM-8**	T1	1 (8%)	1 (8%)	1.0 ^4^	2 (8%)
	T2	6 (50%)	7 (58%)		13 (54%)
	T3	3 (25%)	2 (17%)		5 (21%)
	T4	2 (17%)	2 (17%)		4 (17%)
**Nodal status TNM-8**	N0	0 (0%)	0 (0%)	1.0 ^5^	0 (0%)
	N1	12 (100%)	11 (92%)		23 (96%)
	N2	0 (0%)	1 (8%)		1 (4%)
	N3	0 (0%)	0 (0%)		0 (0%)
**Metastasis TNM-8**	M0	12 (100%)	12 (100%)	1.0 ^6^	24 (100%)
	M1	0 (0%)	0 (0%)		0 (100%)
**AJCC TNM-8**	I	7 (58%)	8 (67%)	1.0 ^7^	15 (63%)
	II	3 (25%)	2 (17%)		5 (21%)
	III	2 (17%)	2 (17%)		4 (17%)
	IV	0 (0%)	0 (0%)		0 (0%)
**Response**	CR	0 (0%)	12 (100%)	<0.001 ^8^	12 (50%)
	PR/NR	12 (100%)	0 (0%)		12 (50%)
**Smoking**	Ever	10 (83%)	10 (83%)	1.0 ^9^	20 (83%)
	Never	2 (17%)	2 (17%)		4 (17%)
**WHO status**	0	12 (100%)	12 (100%)	1.0 ^10^	24 (100%)
	1	0 (0%)	0 (0%)		0 (0%)
	2	0 (0%)	0 (0%)		0 (0%)
	3	0 (0%)	0 (0%)		0 (0%)

^1^ CR = Complete response; PR/NR = Partial and non-response respectively. ^2^ Fischer exact test. ^3^ Student’s *t*-test. ^4^ Fischer exact test T1–2 vs. T3–4. ^5^ Fischer exact test N0–1 vs. N2. ^6^ Fischer exact test. ^7^ Fischer exact test I–II vs. III–IV. ^8^ Fischer exact test CR vs. PR/NR. ^9^ Fischer exact test. ^10^ Fischer exact test status 0–1 vs. 2–3.

**Table 2:** Patients included in the validation cohorts and their tumour characteristics.

		Patients with HPV+ TSCC/BOTSCC Included in qPCR RNA Validation Cohort	Patients with HPV+ TSCC/BOTSCC Included in IHC Protein Validation Cohort	*p*-Value
**Sex**	Male	37 (79%)	30 (68%)	0.3 ^1^
	Female	10 (21%)	14 (32%)	
**Age**	Mean	60.0 years	61.0 years	0.6 ^2^
	Min	42 years	33 years	
	Max	90 years	85 years	
**Tumour size TNM-8**	T1	14 (30%)	12 (27%)	0.3 ^3^
	T2	18 (38%)	13 (30%)	
	T3	12 (26%)	5 (11%)	
	T4	3 (6%)	14 (32%)	
**Nodal status TNM-8**	N0	4 (9%)	6 (14%)	0.5 ^4^
	N1	37 (79%)	23 (52%)	
	N2	4 (9%)	14 (32%)	
	N3	2 (4%)	1 (2%)	
**Metastasis TNM-8**	M0	47 (100%)	43 (98%)	0.5 ^5^
	M1	0 (0%)	1 (2%)	
**AJCC TNM-8**	I	28 (60%)	18 (41%)	<0.0001 ^6^
	II	14 (30%)	6 (14%)	
	III	5 (11%)	14 (32%)	
	IV	0 (%)	6 (14%)	
**Response**	CR	34 (72%)	33 (75%)	0.8 ^7^
	PR/NR	13 (28%)	11 (25%)	
**Smoking**	Ever	29 (62%)	25 (57%)	0.7 ^8^
	Never	18 (38%)	19 (43%)	
**WHO status**	0	45 (96%)	40 (91%)	1.0 ^9^
	1	2 (4%)	4 (9%)	
	2	0 (0%)	0 (0%)	
	3	0 (0%)	0 (0%)	

^1^ Fischer exact test. ^2^ Student’s *t*-test. ^3^ Fischer exact test T1–2 vs. T3–4. ^4^ Fischer exact test N0 vs. N1–3. ^5^ Fischer exact test. ^6^ Fischer exact test I–II vs. III–IV. ^7^ Fischer exact test CR vs. PR/NR. ^8^ Fischer exact test. ^9^ Fischer exact test status 0–1 vs. 2.

## Data Availability

Due to privacy concerns with expression data, processed, deidentified data will be made available upon reasonable request to the corresponding author.

## Supplementary Materials

The following supporting information can be downloaded at: https://www.mdpi.com/article/10.3390/cancers15071954/s1, Table S1: Significant differences in the gene expression between tumours from patients with favourable prognosis and those with poor survival.
